# Prevalence of Osteoporosis in Patients with Distal Radius Fracture from Low-energy Trauma: An Observational Study

**DOI:** 10.31729/jnma.8884

**Published:** 2025-02-28

**Authors:** Manoj Kandel, Sarik Kumar Shrestha, Krishna Prasad Paudel, Sunil Panta, Deepak Banjade, Ramesh Syangtan, Sushil Thapa

**Affiliations:** 1 Department of Orthopedics and Trauma Surgery, Bharatpur Hospital, Bharatpur, Chitwan, Nepal

**Keywords:** *bone density*, *distal radius fracture*, *osteoporosis*, *osteoporotic fracture*

## Abstract

**Introduction:**

Osteoporosis is characterized by low bone mass, leading to enhanced bone fragility. Low-energy fractures of distal radius are related to osteoporosis and is therefore related to increased risk of subsequent hip fractures. The objective of this study was to study the prevalence of osteoporosis in patients with distal radius fracture from low-energy trauma. This will emphasise the need to investigate these patients for osteoporosis and make practitioners should be aware of the possibility of osteoporosis in such patients.

**Methods:**

This observational cross-section study evaluated the prevalence of osteoporosis in patients with distal radius fracture from low-energy trauma among patients presenting to tertiary level hospital of Nepal from October 2022 to September 2023. The participants' demographic data, mechanism of injury, BMD values and T-score values were recorded and diagnosis of osteoporosis and osteopenia was made according to WHO diagnostic criteria. Ethical approval was taken from Institutional Review Committee (Reference Number: 079/80-015).

**Results:**

The study involved 96 patients with mean age of 68.27±4.09 years. The mean BMD among the participants was 0.69±0.02 and the mean T-score was -2.75±0.23. Diagnosis of osteoporosis was made in 88 (91.67%) patients and diagnosis of osteopenia was made in 8 (8.33%) patients based on the T-score using the WHO criteria. Of the 88 (91.67%) patients who met the diagnosis of osteoporosis, only 45 (46.88%) patients had started treatment with bisphosphonates for osteoporosis.

**Conclusions:**

We have found a high prevalence of osteoporosis among patients with low-energy distal radius fracture.

## INTRODUCTION

Osteoporosis is characterized by low bone mass, leading to enhanced bone fragility.^1^ The global burden of osteoporosis is substantial, with prevalence increasing with increasing age.^2^ Osteoporosis is still under-diagnosed in regions with limited access to diagnostic tools such as dual-energy X-ray absorptiometry (DEXA) scans.^3,4^

Osteoporosis results in low-energy fractures commonly in the spine, hip and distal radius.^5-10^ Practitioners should be aware of the possibility of osteoporosis in such patients.^11^ Understanding the epidemiology of osteoporosis in Nepal is crucial for developing effective prevention and treatment strategies.

We aim to find the prevalence of osteoporosis in patients with low-energy distal radius fractures. Since there is limited availability of DEXA scan in many regions of Nepal, this study will help us to understand if patients with low-energy distal radius fracture should be investigated for osteoporosis and if they should receive treatment for osteoporosis even without DEXA scan.

## METHODS

This is a observational cross-section study performed to study the prevalence of osteoporosis in patients with distal radius fracture from low-energy trauma. The study was conducted from October 2023 to September 2024 in a tertiary level hospital of Nepal. The study included all the patients with distal radius fracture who had undergone DEXA Scan within the study duration.

The study was started after obtaining ethical approval was obtained from Institutional Review Committee (IRC) of Bharatpur Hospital (Reference Number: 079/80-015) before commencing the study. All the patients with aged more than 50 years with distal radial fractures from low-energy trauma who had undergone DEXA scan after fracture management and with available bone mineral density (BMD) and T-score results were included in the study. The patients who had taken bisphosphonates before being included in the study were excluded from the study. Written informed consent was obtained before enrolment in the study. Patients with a fracture of distal radius occurring after high-energy trauma and patients with known secondary osteoporosis, as associated with chronic steroid use and hyperparathyroidism were excluded from the study. Patients with rheumatoid arthritis and patients who had taken bisphosphonates in the past before sustaining the fracture were also excluded from the study.

The participants' demographic data, mechanism of injury, BMD values and T-score values were recorded. The diagnosis of osteoporosis and osteopenia was made according to WHO diagnostic criteria.^12-14^ Patients with T-score of -1 and above are classified as normal, those with T-score between -1 and -2.5 are classified has having osteopenia and those with T-score of -2.5 and below are classified as having osteoporosis.^13-14^ The treatment that the patient had received for osteoporosis after presenting to the hospital and before obtaining the results of DEXA scan was also recorded. Fracture type according to AO classification and treatment received for distal radius fracture was also recorded. The data collected was entered in Microsoft Excel and data analysis was done using SPSS version 24. Mean and standard deviation were used for continuous data and percentages for categorical data. The patients' data have been stored in a password-protected computer and patients' personal information have been kept confidential.

## RESULTS

The study involved 96 patients presenting with distal radius fracture from low-energy trauma such as fall from standing height for whom DEXA scan was done.

The age of participants included in the study ranged from 60 to 75 years with mean age of 68.27±4.09 years. There were 47 (48.96%) male patients and 49 (51.04%) female participants (Table 1).

**Table 1:** Patient demographics (n=96).

Patient demographics	Mean±SD/Number (Percentage)
Age	68.27±4.09
BMD	0.69±0.02
T-score	-2.75±0.23
**Sex**	
Male	47 (48.96)
Female	49 (51.04)

**Table 2:** Diagnosis and treatment received (n=96).

Diagnosis and treatment	n (%)
**Diagnosis**	
Osteoporosis	88 (91.67)
Osteopenia	8 (8.33)
**Treatment received in patients with osteoporosis**	
Calcium, Vitamin D, Bisphosphonates	45 (51.14)
Calcium, Vitamin D	43 (48.86)
**Treatment received in patients with osteopenia**	
Calcium, Vitamin D, Bisphosphonates	0 (0)
Calcium, Vitamin D	8 (100)

The BMD of the participants ranged from 0.65 to 0.74 with mean BMD 0.69±0.02. The T-score ranged from -2.30 to -3.30 with mean T-score of -2.75±0.23. Diagnosis of osteoporosis was made in 88 patients and diagnosis of osteopenia was made in 8 patients based on the T-score values using the WHO criteria for osteoporosis (Table 2). Of the 47 male patients, 43 patients met the diagnostic criteria for osteoporosis and 4 patients met the diagnostic criteria for osteopenia. Of the 49 female patients, 45 patients were diagnosed with osteoporosis and 4 patients were diagnosed as having osteopenia.

Of the 88 patients who met the diagnosis of osteoporosis, only 45 patients had started treatment with bisphosphonates, calcium and vitamin D for osteoporosis before the results of DEXA scan were available while 43 patients only received treatment with calcium and vitamin D supplements apart from treatment of the fracture (Table 2). Of the 8 patients with osteopenia, all the patients had received treatment with calcium and vitamin D supplementation and none of these patients had received bisphosphonates (Table 2).

Of the 96 distal radius fractures included in our study, 66 (68.75%) were AO type A (extra-articular), 10 (10.42%) were AO type B (partial articular), and 20 (20.83%) were AO type C (complete articular). Regarding fracture treatment, 56 (58.33%) fractures were treated conservatively with application of Plaster of Paris cast, 38 (39.58%) fractures were treated with closed reduction and percutaneous pinning (CRPP) with K-wires, and 2 (2.09%) fractures were treated with open reduction and internal fixation (ORIF) with distal radius volar locking plate.

## DISCUSSION

In this study, we evaluated 96 patients with low-energy distal radius fracture presenting to Orthopaedics OPD of a tertiary centre of Nepal. The current recommendations for screening of osteoporosis include all women 65 years and older, selected postmenopausal women and men 50 to 69 years of age with risk factors for fracture and all men 70 years and older.^15,16^ According to these recommendations, all patients greater than 50 years of age with risk for fracture or history of fracture from low-energy mechanism must be screened for osteoporosis.^15,16^ Therefore, all patients with low-energy distal radius fractures meet the criteria for screening of osteoporosis.

The mean age of patients in our study was 68.27±4.09 years and the study included 48.96% male and 51.04% patients with distal radius fracture. Previous studies have shown that distal radius fractures are more common in females in patients above 50 years of age due to post-menopausal osteoporosis.^17-18^ According to a study by Koo et al., the peak incidence of distal radius fractures in females occurred in the perimenopausal age group, whereas the incidence for males peaked between age of 30 to 50.^19^ In contrast to these studies, we have seen almost equal distribution of distal radius fractures in both sexes in our study. We postulate that this may be because elderly females in our part of the world tend to be less physically active compared to males and are less likely to suffer falls or injuries. It can also be due to the fact that females are less likely to seek medical attention for minor falls.

The mean BMD was 0.69±0.02 and the mean T-score was -2.75±0.23 in this study. The BMD and T-score in our study was lower than that found in similar studies evaluating these values in patients with distal radius fracture.^20-22^ A study by 0yen et al. compared the T-scores in patients with distal radius fracture with age-matched controls without fracture.^21^ This study showed the mean T-score in patients with distal radius fracture to be -1.65, and they found the T-score in patients with distal radius fracture to be significantly lower compared to controls. The mean BMD in patients with distal radius fracture another study by Egund et al. was found to be 0.929±0.14.^22^ The lower value of BMD and T-score in our study may be because of inclusion of only patients above 50 years of age in our study. Another possible reason may be that in our part of the world, patients are not regularly evaluated for osteoporosis and patients usually do not receive any preventive treatment for osteoporosis.

Among the patients who participated in the study, 91.67% were osteoporotic and 8.33% were osteopenic. The prevalence rate of osteoporosis in our study was much higher than the rates in other countries. The prevalence of osteoporosis in postmenopausal women with distal radius fracture in Germany was found to be 43% in a study by Endres et al.^9^ Another study by Jung et al. evaluating the bone mineral density and prevalence of osteoporosis in postmenopausal Korean women with low-energy distal radius fractures found the prevalence of osteoporosis to be 52% in these patients.^10^ The prevalence of osteoporosis was found to be 59.50% and the prevalence of osteopenia was found to be 29.10% among patients above 50 years of age with low-energy distal radius fracture in Thailand.^23^ A similar study from Sweden reported the prevalence of distal radius fracture to be 37% while another study done in Norway reported the prevalence as 34%.^21,24^ The higher prevalence of osteoporosis seen in our study may be related to under diagnosis of osteoporosis in our part of the world than in more developed countries, due to which only a small number of patients are treated for osteoporosis. This would result in a higher number of patients suffering from osteoporotic fractures, including distal end radius fractures.^3,25^

Although the prevalence of osteoporosis in our study was high, patients with such fractures were rarely investigated for osteoporosis prior to this study. Among all participants, none of the patients had undergone prior evaluation of BMD. In Korea, only 8.70% of women with wrist fractures had a BMD study.^4^ Similarly, in the United Kingdom, only 8% patients were undergoing treatment for osteoporosis at the time of their wrist fracture.^26^ The results from our study and those from other countries emphasise the need to investigate patients with distal radius fracture from low-velocity trauma for osteoporosis even if the fractures are considered minor. It appears that many doctors recognise the need for investigating osteoporosis in more severe fractures like vertebral and hip fractures.^11^ However, increased awareness is needed to investigate fractures resulting from low-energy trauma such as distal radius fracture since osteoporosis rates are high in this group. Also, these fractures may predict the future risk for more sever fractures such as hip fractures.^27^

Of the 96 distal radius fractures included in our study, 66 (68.75%) were AO type A (extra-articular), 10 (10.42%) were AO type B (partial articular), and 20 (20.83%) were AO type C (complete articular). A study from Sweden also reported that majority of distal radius fractures are AO type A (extra-articular) and intra-articular fractures are mostly due to high velocity injuries.^28^ Another study from Singapore also mentions that AO type A distal radius fractures are the most common followed by AO type C and AO type B fractures are the least common.^29^ They have postulated that AO type B fractures are least common because for a partial articular fracture to occur, a specific force with direct impact onto radius is needed and such direct impact occurs less commonly than fall on outstretched hand.^29^ The findings of our study also align with these studies.

Among 96 distal radius fractures, 56 (58.33%) fractures were treated conservatively with application of Plaster of Paris cast, 38 (39.58%) fractures were treated with closed reduction and percutaneous pinning (CRPP) with K-wires, and 2 (2.09%) fractures were treated with open reduction and internal fixation (ORIF) with distal radius volar locking plate. A study done in Singapore also showed that most distal radius fractures are treated conservatively.^29^ They also noted that distal radius fractures in elderly population are more likely to be treated conservatively and they also noted that surgical treatment is more commonly done for intra-articular fractures.^29^

We acknowledge that there are some limitations in our study. Firstly, this was a single-centre study conducted in a tertiary hospital of central Nepal. Further multicentre studies may help to generate more information on the topic. Other limitation of our study is the lack of control group. Further studies comparing the BMD of patients with such fractures and normal population may help to understand more about the prevalence of osteoporosis in Nepalese population. Nevertheless, our study has shed light on the need to evaluate for presence of osteoporosis in distal radius fractures resulting from low-energy trauma even though these fractures are considered minor. There is growing evidence to suggest that the personal and public health burden due to distal radius fracture may have been underestimated.^30^ We also recommend clinicians to be aware of the possibility of osteoporotic fracture in patients presenting with any fracture from low-velocity trauma.

## CONCLUSIONS

We have found a high prevalence of osteoporosis among patients with low-energy distal radius fracture. Therefore, we recommend that all patients who suffer fractures of the distal radius from low-energy trauma should undergo evaluation for osteoporosis and receive treatment if needed in order to prevent future risk of fractures.
